# The threat to our human rights: The repeal of the Human Rights Act 1998

**DOI:** 10.1177/00258024221111908

**Published:** 2022-07-05

**Authors:** Paul Arnell

**Affiliations:** Law School, Robert Gordon University, UK

Human rights were introduced into the United Kingdom law over two decades ago. They were, it was said, brought home.^1^ The Human Rights Act 1998 (HRA) created binding and justiciable human rights for everyone within the jurisdiction of the country. UK courts became the venues for arguments based on human rights for the very first time. The HRA gave effect within UK law to the Council of Europe’s European Convention on Human Rights 1950 (ECHR). Human rights were brought home in the sense that British lawyers and politicians played an important part in developing and writing the ECHR.

The original reasons put forward in 1997 in favour of the incorporation of the ECHR into UK law have stood the test of time. Perhaps, the most important was the desirability of making the rights within the treaty directly accessible to everyone within the jurisdiction of the UK. The cost in resources and time of taking a case to the European Court of Human Rights (ECtHR) in Strasbourg would generally be greatly reduced where human rights were arguable in the UK courts. Human rights would become a part of the UK law and jurisprudence, shaped by British judges, and available to everyone. This is what has happened.

The HRA gives effect to the rights in the ECHR by way of a nuanced and considered balance between differing and at times competing considerations. Central amongst them are the human rights of individuals and the UK's public interest, and the identity and particularities of the UK and pan-European human rights jurisprudence. Further, the HRA respects Parliamentary sovereignty whilst allowing the courts to consider and adjudge legislation and the actions of public authorities, including the Government itself.

The system of human rights protection created by the HRA has been a success, and is widely considered as such.^2^ Cases where human rights violations have been found include where mentally incapacitated persons have been deprived of liberty without authorisation, where a policy of the blanket retention of innocent people's DNA has been followed and where there was opacity over the reviewability of life sentences in England and Wales. Regrettably, the repeal of the HRA appears imminent and with it the delicate balance of considerations it created will come to an end. The Johnson Government's plan for a British Bill of Rights to replace it was intimated in the Queen's speech on 10 May 2022.

The Government's arguments and proposals for a British Bill of Rights are set out in broad terms in a Consultation Paper published in March 2022.^3^ It follows in time, but not in substance, the Government's own independent review of the operation of human rights law in the UK.^4^ The Government is set on addressing the problems it perceives with the law in spite of the Review concluding that the law was in general working well, and in the face of widespread objection and concern by both governmental and non-governmental organisations.^5^

There are a number of reasons why the proposed changes to the system of human rights protection proposed are unwelcome. They are linked by the fact that the amendments to the law will lessen the current level of human rights protection. Notably, this will occur not through a reduction in the number of applicable rights but instead through changes to the procedures and rules governing how they may be vindicated. The important balances under the HRA will accordingly be tilted in favour of the Government and the state at the expense of the individual and the courts.

## The balance between the individual and the Government

The essence of human rights protection entails empowering individuals to lawfully act against the state when their entitlements are violated or threatened seeking an injunction, damages or another remedy or combination of remedies. Amongst the important rights applicable in the UK are those prohibiting inhuman and degrading treatment and guaranteeing the right to liberty and respect for private and family life. Most human rights have certain legitimate limits or qualifications. They are weighed against other considerations, such as the prevention of disorder and crime. The courts have developed the law and created tests under which the competing interests are balanced. The Consultation Paper suggests changes to the law that shift the balance in the direction of the Government at the expense of the individual. This takes place through the introduction of a permission stage for human rights claims, decoupling human rights claims from other private law remedies such as negligence, and restraining the imposition and expansion of positive human rights obligations.^6^ Together with other changes, the effect will be a weakening of the existing level of human rights protection.

## The balance between the ECtHR and the Government

The HRA obliges UK courts to ‘take into account’ the case law of the ECtHR when considering cases with a human rights relevance. This aspect of the present system has been subject to strident criticism by the Government. This is due to the so-called ‘mission-creep’ of the ECtHR and, in particular, the interpretation of the phrase ‘take into account’ by the UK courts.^7^ This has led, in the Government's view, to an interpretation of human rights that has stymied public authorities in carrying out public policy. In fact, the relationship between the ECtHR and UK courts is a facet of the incorporation of the ECHR into domestic law and the obligation within it to secure everyone within its jurisdiction the rights and freedoms within it. The Consultation Paper argues, in essence, that the effect of the ECtHR case law has been too great, and that domestic precedent and other authorities should have a greater role. There is no doubt, however, that an important feature of adherence to the ECHR is supranational scrutiny and the input of the ECtHR's jurisprudence into national law and practice. Further, and countering the Government's view, the approach taken by the UK courts has evolved in recent years. The degree of judicial adherence to Strasbourg jurisprudence has been lessened. The proposed changes in this vein, tilting the balance in favour of the Government, are accordingly unnecessary.

## The balance between UK courts and the Government

The HRA requires courts to interpret legislation, as far as is possible to do so, compatibly with human rights. Where this is not possible, a court may make a declaration of incompatibility. A declaration does not, however, affect the validity of Acts of the UK Parliament. In attempting to meet the interpretive obligation in the HRA courts have at times taken an expansive approach to the meanings of words. This has caused concern in Governmental circles. The Consultation Paper proposes to limit the interpretive obligation upon the courts, in order to afford greater judicial respect to the will of Parliament in enacting legislation. Again, this change tilts the balance under the HRA in favour of the Government. Its ability to pass legislation through Parliament, where it has a majority, will in essence have fewer constraints.

## The bigger picture

The HRA is the centrepiece of UK human rights law. Its repeal and replacement are designed to increase the leeway the Government has in carrying out its policies. A concomitant will be a lessening of the protection of individuals within and across the UK. The HRA, though, exists within a matrix of international and national law affecting human rights. Changes to the human rights system will take place in an established context.

The first and most important point here is that the UK Government has promised to remain bound in international law to the ECHR itself. Continued membership is indeed crucial. One facet of which is the obligation upon the UK to adhere to the ECHR under the terms of the Trade and Cooperation Agreement 2020 governing its relationship with the EU. More generally, withdrawal from the ECHR would be a hugely retrograde step for individuals within the country and as regards the UK's international standing. From being instrumental in the conclusion of the ECHR, then ‘bringing rights home’ to repudiating the treaty – such a development is beyond reason.

The second point is that within the UK the devolved administrations are moving in the contrary direction. Of note here are the Scottish Government's efforts to incorporate further rights into Scots law. The Convention on the Rights of the Child 1989 and the UN Convention on Economic, Social and Cultural Rights 1966 are amongst the agreements it is working to bring into law. The obvious questions arising are why is there such a difference in views between the UK and Scottish Governments and which is the preferable direction of travel?

Finally, the UK is one of the few countries in the world without a single written constitution that delimits the powers of the main institutions of the state and contains a set of standards to which both the government and citizens must adhere. Ultimate power and authority are instead held by the UK Parliament. It is sovereign. A majority Government is able, through Parliament, to legislate with little relatively little constraint. The HRA facilitates a welcome, indeed necessary, degree of judicial scrutiny on Government and Parliament. There are weighty arguments that the limits upon and scrutiny of the Government should be increased not lessened. The present proposal to repeal the HRA is indeed a threat to our human rights.

## Notes

1. The White Paper leading to the change in the law was titled ‘Rights Brought Home: The Human Rights Bill’, Cm 3782, October 1997, cited at https://assets.publishing.service.gov.uk/government/uploads/system/uploads/attachment_data/file/263526/rights.pdf.

2. See, for example, Gearty, C., The Human Rights Act Comes of Age, (2022) 2 European Human Rights Law Review 117–126 and Foster, S., Should it Go or Should it Stay? The Coming of Age of the Human Rights Act 1998, or Time to Say Goodbye?, (2021) 26(2) Coventry Law Journal 23–39.

3. Human Rights Act Reform: A Modern Bill of Rights – Consultation, cited at https://www.gov.uk/government/consultations/human-rights-act-reform-a-modern-bill-of-rights/human-rights-act-reform-a-modern-bill-of-rights-consultation.

4. Independent Human Rights Act Review, December 2021, cited at https://www.gov.uk/guidance/independent-human-rights-act-review.

5. The Scottish Human Rights Commission has said that the proposals should be a real concern for all who value human rights and the rule of law, cited at https://www.scottishhumanrights.com/projects-and-programmes/defending-the-human-rights-act/. The NGO Liberty describes the plans as a ‘power grab’ by the Government, at https://www.libertyhumanrights.org.uk/fundamental/stand-up-to-power/.

6. These proposals are set out in Part II of the Consultation, supra note 3, at paras 218–231.

7. As to the former see Amos, M., The Value of the European Court of Human Rights to the United Kingdom, (2017) 28(3) European Journal of International Law 763–785, and the latter Graham, L., Taking Strasbourg Jurisprudence into Account, (2022) 2 European Human Rights Law Review 163–172.

